# Combined Transcriptome and Metabolome Analyses Provide New Insights into the Changes in the Flesh Color of Anthocyanins in Strawberry (*Fragaria* × *ananassa* (Weston) Duchesne ex Rozier)

**DOI:** 10.3390/genes15111391

**Published:** 2024-10-29

**Authors:** Xiangrong Ren, Meile Sun, Jingtao Hui, Jing Yang, Jun Zhang, Pengbing Li, Guocang Lin

**Affiliations:** Comprehensive Experimental Field, Xinjiang Academy of Agricultural Sciences, Urumqi 830091, China; renxiangrong@xaas.ac.cn (X.R.); sunmeile@xaas.ac.cn (M.S.); huijingtao@xaas.ac.cn (J.H.); yangjing@xaas.ac.cn (J.Y.); zhangjun@xaas.ac.cn (J.Z.); lipengbing@xaas.ac.cn (P.L.)

**Keywords:** strawberry, flesh color, RNA-seq, metabolomics

## Abstract

Background: Strawberries are bright in color, sweet and sour in taste, and rich in nutrients and flavonoid compounds such as anthocyanins and proanthocyanidins. The synthesis and accumulation of anthocyanins are the decisive factors that make strawberries appear bright red. From the perspective of plant breeding, a change in flesh color is an important goal. Methods: In this study, two strawberry plants with different flesh colors were selected, and transcriptome and metabolome analyses were performed during the color change period (S1) and ripening period (S2). Results: RNA-seq revealed a total of 13,341 differentially expressed genes (DEGs) between and within materials, which were clustered into 5 clusters. A total of 695 metabolites were detected via metabolome analysis, and 243 differentially regulated metabolites (DRMs) were identified. The anthocyanin biosynthesis, starch and sucrose metabolism and glycolysis/gluconeogenesis pathways were determined to be important regulatory pathways for changes in strawberry flesh color through a joint analysis of RNA-seq data and the metabolome. The leucoanthocyanidin reductase (LAR) and chalcone synthase (CHS) gene is a key gene related to anthocyanins, cinnamic acid, and phenylalanine. In addition, through joint RNA-seq and metabolome analyses combined with weighted gene co-expression network analysis (WGCNA), we identified 9 candidate genes related to strawberry flesh color. Conclusions: Our research findings have laid the groundwork for a more comprehensive understanding of the molecular mechanisms governing the color transformation in strawberry flesh. Additionally, we have identified novel genetic resources that can be instrumental in advancing research related to strawberry color change.

## 1. Introduction

The strawberry (*Fragaria* × *ananassa* Duch.) is a perennial herbaceous plant in the Rosaceae family that is widely cultivated worldwide [1]. Its fruit is bright in color, sweet and sour in taste, and rich in nutrients (vitamin A, vitamin C, anthocyanins, and proanthocyanidins, etc.), and it is very popular among consumers and growers [2]. Anthocyanins are not only important antioxidants that have important health functions in the human body but also the main reason for the color of strawberries [3]. More importantly, anthocyanins have a greater antioxidant capacity than vitamin C, vitamin E, etc.; they can effectively remove free radicals, and they have important effects, delaying human skin aging, whitening the skin, protecting eyesight, providing antibacterial and anti-inflammatory effects, and preventing cancer and cardiovascular/cerebrovascular diseases [4]. Fruit color is the core indicator for judging its appearance quality and directly affects its commercial value [5]. In China, strawberries are cultivated primarily in winter and spring. The temperature of the cultivation environment is high, but the light is insufficient, which leads not only to rapid development of strawberries, insufficient sugar accumulation, and high acidity but also to poor color and low commercial value [6]. Studies have shown that light has a significant mediating effect on the synthesis and accumulation of anthocyanins in fruits. Strawberry plants can use light to photosynthesize sugars and other substances, thereby providing an energy source for the synthesis of anthocyanins and maintaining osmotic pressure in cells [7]. Moreover, as an external signal, light directly regulates the expression of genes with light-responsive elements in the anthocyanin biosynthesis pathway. Low temperatures can induce the accumulation of anthocyanins, whereas high temperatures accelerate the degradation of anthocyanins [8]. The response of cells to temperature is complex, and the response of fruits to temperature during anthocyanin synthesis and its molecular mechanism are not yet clear. The effective regulation of sugar, acid, and anthocyanin metabolism in strawberries has always been an important issue that strawberry growers are committed to solving [9]. Therefore, an in-depth analysis of the regulatory mechanism of anthocyanin metabolism during the development and ripening of strawberries is crucial for determining strawberry fruit quality [10].

Transcriptome sequencing technology is a type of high-throughput sequencing technology based on assessment at the RNA level that can analyze the transcript information of any part and any state of a species at the single nucleotide level [11]. RNA-seq has high sequencing throughput, a wide application range, and good sensitivity. This tool simplifies the process of analyzing differential expressions of various transcripts, making it easier to identify key differentially expressed genes and factors during gene screening [12]. As the terminal products of cell regulation processes, metabolites describe the phenotypic changes of biological systems well, but the analysis of metabolite diversity and genetic mechanisms is relatively weak [13]. With the completion of plant metabolite databases, metabolomics has often been combined with transcriptomics and other -omics methods in the postgenomic era to conduct comprehensive and in-depth analyses of biological systems. The joint analysis of transcriptomes and metabolomes can provide accurate information on the interactions between genes and metabolites and help researchers construct a regulatory network of the corresponding metabolites, promoting the analysis of gene functions and metabolic pathways [14]. The integrated analysis of metabolomes and transcriptomes is a powerful approach for elucidating gene functions, commonly employed to uncover the genetic and regulatory mechanisms governing plant metabolites. For example, the flavonoid metabolic regulatory networks of maize, kiwifruit, *Ficus carica*, asparagus, potato, ginkgo, and pepper were studied using combined metabolome and transcriptome analyses [15,16,17,18,19]. Transcriptome and metabolome analyses of maize revealed that the accumulation of flavonoids caused the different colors of corn kernels in groups R (red), Y (yellow), and P (purple), and the expression levels of key flavonoid synthesis-related genes were consistent with the accumulation patterns of metabolites in these differently colored corn kernels. The contents of three flavonols (kaempferol-3-rhamnoside, L-epicatechin, and chloranthin) and one dihydrochalcone (phloretin) were the most different in kiwifruit roots and fruits, and genes related to flavonols and dihydrochalcone were highly expressed in the roots. In addition, *AcRAP2-4*, *AcAP2-4*, and *AcbHLH62* are key candidate genes that control the accumulation of flavonoids in kiwifruit. Based on the transcriptome and target metabolome data of four differently colored pepper varieties, the metabolic regulatory network of pepper flavonoids was constructed, and the key genes were revealed [19]. The transcriptomes of three *Solanum tuberosum* buds were analyzed in conjunction with the anthocyanin metabolites. The results revealed that 22 anthocyanins were strongly correlated with 119 genes related to flavonoid metabolism, hormone synthesis, transcriptional regulation, and signal transduction [20]. Through the combined analysis of RNA-seq and metabolome data from *Dianthus* species, 49 betaines were identified, and the *CYP76AD15* and *MjHCGT* genes were determined to be involved in the hydroxylation of tyrosine and the formation of (hydroxy)cinnamoyl-glucose esters during the betaine synthesis process. In addition, the lack of anthocyanins in *Mirabilis jalapa* was found to be due to the deletion of a partial sequence in the region of the active site for the anthocyanin synthesis gene (ANS) [21]. Through solid-phase extraction and GC–MS, 85 volatile substances were detected in 23 *Cucumis sativus* tissues; combined with RNA-seq and in vitro biochemical experiments, terpene synthase 11 (*TPS11*)/*TPS14*, *TPS01*, and *TPS15* were demonstrated to be responsible for the synthesis of terpenes [22]. Through an analysis of the RNA-seq and metabolome of *Arabidopsis* strains along with the transgenic rice *Os-LBD37*/*ASL39* genes, the content of nitrogen-containing metabolites in the transgenic strains changed significantly; thus, *Os-LBD37*/*ASL39* was identified as a metabolism regulatory gene of rice nitrogen [23].

The strawberry is a widely cultivated fruit worldwide and is loved by growers and consumers. The global strawberry planting area increased from 32.18 million mu in 2019 to 37.70 million mu in 2023, representing an increase of 16.27%, and the output was 19.17 million tons (which is an increase of 35.25%) (https://www.sgpjbg.com/info/4d0dd412f47b19743b69cbf7337a2093.html (accessed on 10 July 2023)). As the largest strawberry producer, China has a strawberry planting area of more than 2 million mu and a production volume of nearly 4 million tons. Fruit color is the core indicator for determining fruit appearance quality. The color is the result of anthocyanin accumulation and has an important impact on the economic value of the fruit. Anthocyanins are important products of plant flavonoid metabolism and have a strong antioxidant capacity. The antioxidant capacity of anthocyanins is 20 times greater than that of vitamin C and 50 times greater than that of vitamin E [24]. As natural antioxidants with high scavenging capacity, anthocyanins are used to resist lipid peroxidation, protect cell membranes, prevent and treat cardiovascular diseases, and reduce capillary permeability. After the consumption of anthocyanins for days, the activities of superoxide dismutase and glutathione peroxidase in human blood are significantly greater than those before and after consumption [25]. In an experimental study on exhaustive exercise in mice with 1% kg/kg body weight of anthocyanins, the swimming time of the drug-treated group was significantly longer than that of the control group, and the activity of superoxide dismutase in liver tissue was greater than that of the control group. These compounds not only have important health functions for the human body but also affect the intrinsic quality of strawberries [26]. Increasing the anthocyanin content of strawberries has become one of the goals of plant breeding. While the key enzyme-encoding genes and regulatory factors in the anthocyanin synthesis pathway have been extensively researched, much of the focus has been on known structural genes and the three primary transcription factors—MYB, bHLH, and WD40 [27,28]. Through joint analysis of the transcriptome and metabolome, we can fully understand the changes in gene expression and metabolites during color changes in strawberry flesh and reveal the gene regulatory network involved in anthocyanin synthesis. These findings will help to elucidate the mechanism underlying the formation and regulation of strawberry flesh color, provide a theoretical basis for breeding improvement and cultivation management, and improve economic benefits for growers. In this study, two strawberry varieties (one with red flesh and one with white flesh) were selected, and transcriptome and metabolome analyses were conducted across the color change and maturity stages. Cluster analysis and KEGG enrichment analysis were conducted on both the differentially expressed genes and the differentially abundant metabolites. Subsequently, a regulatory network pertaining to flavonoid synthesis was established. Candidate genes related to strawberry flesh color changes were identified through WGCNA. This study aimed to investigate the variations in anthocyanin content during the color transition of strawberry flesh by integrating transcriptome and metabolome analyses. The objective was to gain fresh perspectives on the color formation mechanism. These findings lay the groundwork for a more in-depth exploration of the mechanisms underlying strawberry flesh color evolution and introduce novel genetic resources for future research on strawberry color alteration.

## 2. Materials and Methods

### 2.1. Plant Materials

**Figure 1:**
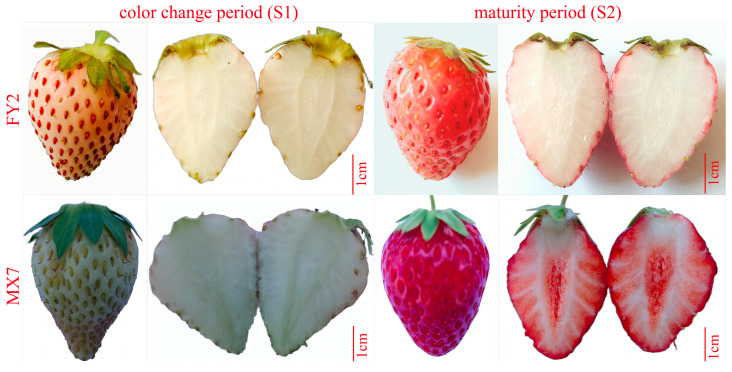
Fruit appearance and flesh color of FY2 and MX7 before and after color change; scale bar = 1 cm.

The strawberry variety Fenyu 2 (FY2, bred by the Hangzhou Academy of Agricultural Sciences) has a pink peel and white flesh. Owing to the strong sunlight in Xinjiang, China, the fruit skin color of FY2 is darker than that in other locations. This variety and Miaoxiang 7 (MX7, bred by the Shandong Academy of Agricultural Sciences, with a red peel and orange-red flesh) were selected and planted in the small berry germplasm resource garden of the Xinjiang Academy of Agricultural Sciences Comprehensive Experimental Field, Xinshi District, Urumqi, Xinjiang. The plot area was 300 m^2^ (100 m × 3 m), with 3 ridges in each plot, which were 30 cm high, 45 cm wide, and 35 cm wide. A planting method involving large ridges and double rows was adopted, with a spacing of 20 cm and a drip irrigation pipe placed in the middle of each ridge. Planting was performed on 5 September 2022, and the fruits matured in May 2023. Sampling was performed 30 days after flowering (color change period, S1) and 35 days after flowering (maturity period, S2; see Figure 1). Pulp (peel and pith removed) tissue was collected from each sample, immediately frozen in liquid nitrogen, brought back to the laboratory, and stored at −80 °C for later use.

### 2.2. RNA-Seq and Analysis

The samples were subsequently transported to Biomarker (Beijing, China) on dry ice for sequencing. Their total RNA was extracted using advanced molecular biology equipment. Following RNA extraction, the purity, concentration, and integrity of the RNA were assessed using a NanoDrop 2000 and an Agilent 2100. Subsequently, a library was generated, and a Qubit 3.0 fluorescence quantification instrument was utilized for preliminary quantification, with a target concentration of 1 ng/µL or greater. The Qsep400 high-throughput analysis system was then employed to check the inserted fragments of the library. Finally, the library’s effective concentration (library effective concentration > 2 nM) was precisely quantified using the Q-PCR method to ensure the library’s quality. After the library quality inspection, PE150 sequencing was performed using the Illumina NovaSeq 6000 (Illumina, San Diego, CA, USA) sequencing platform. The RNA data presented in the study have been deposited in the NCBI repository under accession number PRJNA1157624. Fastp software (version 0.23.4)was used to eliminate reads containing adapters and those of low quality, resulting in the acquisition of clean data [29]. Clean reads were aligned with the strawberry cultivar (Wongyo 3115) reference genome (https://datadryad.org/resource/doi:10.5061/dryad.b2c58pc (accessed on 15 August 2023)) using HISAT2 software (version 2.2.1) , and the reads from the alignment were quantified with StringTie [30]. The fragments per kilobase of transcript per million fragments mapped (FPKM) value was used for normalization and as an indicator to measure the gene expression levels. Differentially expressed genes were identified using edgeR software (version 4.2.2), considering the count values of genes in each sample. A fold change of ≥2 and an FDR < 0.01 were used as the criteria for screening [31]. The differentially expressed genes (DEGs) underwent GO and KEGG enrichment analyses using ClusterProfiler (version 4.12.6) with the hypergeometric test method [32].

### 2.3. Metabolite Extraction and Detection

The freeze-dried sample was weighed (50 mg), and an extraction solution comprising methanol, acetonitrile, and water in a volume ratio of 2:2:1 (1000 μL) was added. The mixture was vortexed for 30 s, after which steel beads were introduced. Subsequently, the mixture underwent grinding at 45 Hz for 10 min and was ultrasonicated for an additional 10 min in an ice-water bath. After standing at −20 °C for an hour, the mixture was centrifuged at 12,000 rpm for 15 min. Subsequently, 500 μL of the supernatant was meticulously transferred and dispensed into an EP tube. The extract was dried using a vacuum concentrator, followed by the addition of 160 μL of extraction solution (acetonitrile: water in a volume ratio of 1:1) for redissolving the dried metabolites. After vortexing for 30 s and ultrasonication in an ice-water bath for 10 min, the mixture underwent centrifugation at 12,000 rpm for 15 min. The resulting supernatant was meticulously removed for analysis on the instrument. The detection was conducted using the Waters Acquity I-Class PLUS ultra-high-performance liquid chromatography system coupled with an AB Sciex Qtrap 6500+ high-sensitivity mass spectrometer. The chromatographic column utilized was an Acquity UPLC HSS T3 column (1.8 µm, 2.1 × 100 mm) sourced from Waters, with an injection volume of 2 µL. The mass spectrometry parameters were configured as follows: electrospray ionization temperature at 550 °C; ion spray voltage of 5500 V (positive ion mode) or −4500 V (negative ion mode); gas settings for ion source gas I (GSI), gas II (GSII), and curtain gas (CUR) were respectively adjusted to 50, 55, and 35 psi; and collision-induced ionization parameters were set to medium.

### 2.4. Metabolome Analysis

Using the GB-PLANT database created by Biomarker, a qualitative analysis of substances was conducted utilizing secondary spectrum information. During the analysis, isotope signals, duplicated signals with K^+^, Na^+^, and NH4^+^ ions, as well as repetitive signals from fragment ions of higher molecular weight substances, were excluded. Metabolite quantification was achieved through multiple reaction monitoring analysis using triple quadrupole mass spectrometry [33]. Following the acquisition of mass spectrometry analysis data from various metabolite samples, the peak areas of all mass spectrometry peaks were adjusted. Subsequently, a principal component analysis (PCA) of each sample was conducted based on the metabolite content data matrix using the R language [34]. Metabolites were categorized, and pathway functional annotations were executed utilizing the KEGG database [35]. A relationship model between metabolite expression and sample categories was constructed using partial least squares regression to predict sample categories. Differentially abundant metabolites were identified based on the criteria of a fold change >2 or <1/2 and *p* value < 0.05 [36].

### 2.5. WGCNA

The WGCNA package in R (version 1.73) was employed for co-expression analysis of DEGs utilizing the dynamic branch cutting method [37]. A weighting coefficient of β = 4 was set when the correlation coefficient reached 0.8. The network was formed utilizing an automatic network construction feature with blockwise modules to derive gene co-expression modules. Modules exhibiting a similarity of 0.75 were amalgamated, with ‘minModuleSize’ set as 30 and ‘Merge Cut Height’ as 0.25 being the criteria. The module’s characteristic vector (ME) and the correlation coefficient between the developmental stages of the two materials were computed. The co-expression network was then illustrated using Cytoscape software (version 3.10.3) [38].

### 2.6. qRT–PCR

Total RNA was extracted using the RNAprep Pure Polysaccharide and Polyphenol Plant Total RNA Extraction Kit (Tiangen, Beijing, China). DNase1 working solution (80 μL) was added to all samples to eliminate genomic DNA (gDNA), followed by incubation at room temperature for 15 min. The RNA concentration and integrity of each sample were assessed using a NanoDrop 2000 spectrophotometer (Thermo Fisher Scientific, Waltham, MA, USA). Reverse transcription was carried out using an M-MLV RTase cDNA Synthesis Kit from TaKaRa to generate cDNA; the cDNA concentration of each sample was approximately 100 ng/uL. Quantitative Real-Time PCR (qRT-PCR) analysis was conducted using a Roche LC480 instrument (Roche, Basel, Switzerland) and SYBR Green (Takara, Kyoto, Japan). The reaction mixture volume was 20 μL. The reaction program included a pre-denaturation step at 95 °C for 30 s, followed by 35 cycles of denaturation at 95 °C for 5 s, annealing at 60 °C for 5 s, and extension at 72 °C for 30 s. The LightCycler480 software (version 1.5) was utilized for extracting and exporting the raw data. Relative quantification was performed using the 2^−ΔΔCt^ method, with *Actin* (GenBank accession number: LOC101313051) serving as the internal reference gene. Each program had three biological replicates for analysis [39]. All primers used in this study are listed in Table S1.

## 3. Results

### 3.1. Overall Analysis of Transcriptome Sequencing Data

**Figure 2:**
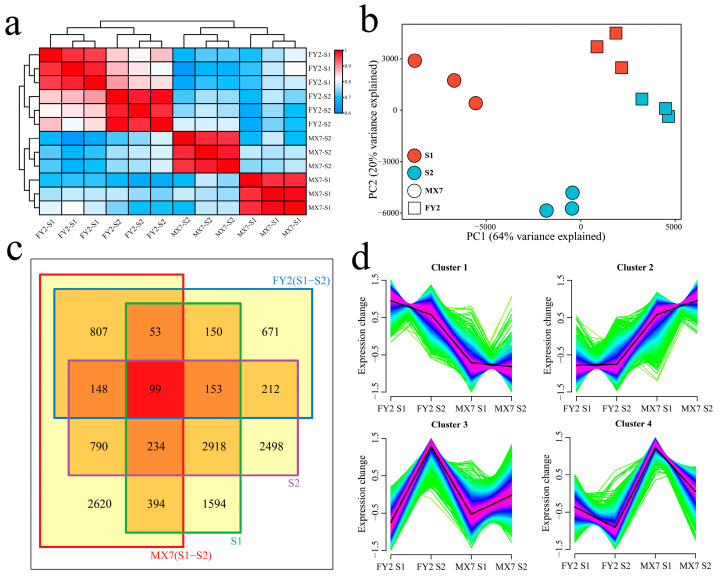
Correlation clustering and differential expression analysis of 12 strawberry RNA-seq datasets. (**a**) Correlation clustering heat map of 12 strawberry RNA-seq datasets; (**b**) PCA of 12 strawberry RNA-seq datasets; (**c**) Venn diagram of DEGs between and within materials; (**d**) k-means clustering line chart of DEGs between and within materials.

The RNA-seq data from 12 samples at two time points during strawberry fruit development yielded 73.45 Gb of clean data, and the clean data from each sample exceeded 5.72 Gb; the percentage of Q30 bases was above 97.21%, and the alignment rate with the reference genome exceeded 92.53% (Table S2). Cluster analysis and PCA were performed on the RNA-seq data of the 12 samples. The correlation coefficient between different biological replicates of the same sample exceeded 0.92. The results of the PCA revealed that the same biological replicate samples were clustered together, indicating that the transcriptome data were reliable and reproducible (Figure 2a,b). The RNA-seq data were divided into two groups: FY2 clustered into one category, and MX7 clustered into another category. The correlation between the two FY2 periods was greater than that between the two MX7 periods, which indicated that the difference between the two MX7 periods was greater than the difference between the two FY2 periods.

Through differential expression analysis, a total of 2293 DEGs were identified during S1 and S2 of FY2, including 671 unique DEGs. A total of 5145 DEGs were identified during S1 and S2 of MX7, including 2620 unique DEGs (Figure 2c). A total of 5595 DEGs were identified in S1 of MX7 and FY2, including 1594 unique DEGs. A total of 7052 DEGs were identified in S2 of MX7 and FY2, including 2498 unique DEGs. This result also revealed that the differences between materials were greater than those within materials and that the differences between materials in S2 were greater than those in S1. Through k-means clustering, 13,341 DEGs were clustered into four clusters (Figure 2d). Cluster 1 exhibited the highest expression level in S1 of FY2, decreasing as fruit development progressed. Cluster 2 displayed the highest expression level in S2 of MX7, with expression levels increasing during fruit development. Cluster 3 showed peak expression in S2 of FY2, with expression levels increasing with fruit development. Cluster 4 had the highest expression level in S1 of MX7, witnessing a decrease in expression as fruit development advanced.

### 3.2. DEG Enrichment Analysis

**Figure 3:**
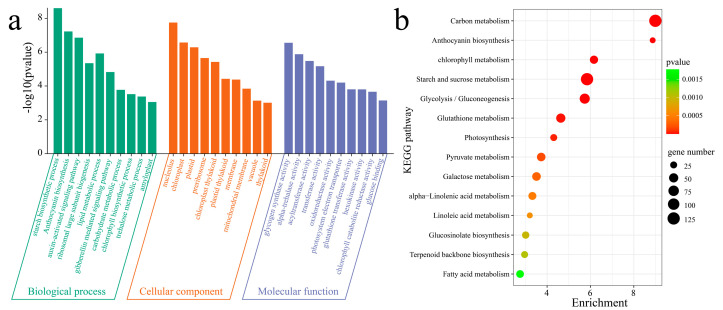
Enrichment analysis of DEGs between and within materials. (**a**) GO enrichment analysis of DEGs between and within materials; (**b**) KEGG enrichment analysis of DEGs between and within materials.

To understand the functions of the DEGs, GO and KEGG enrichment analyses were performed on all the DEGs (13,341) between and within the materials (Figure 3). GO enrichment analysis revealed that for biological processes, there was significant enrichment in the starch biosynthetic process, anthocyanin biosynthesis, auxin-activated signaling pathway, ribosomal large subunit biogenesis, lipid metabolic process, gibberellin-mediated signaling pathway, carbohydrate metabolic process, chlorophyll biosynthetic process, and trehalose metabolic process (Figure 3a). The cellular components that were significantly enriched were the amyloplast, nucleolus, chloroplast, plastid, preribosome, chloroplast thylakoid, plastid thylakoid, membrane, mitochondrial membrane, vacuole, and thylakoid. In terms of molecular function, the activities of glycogen synthase, α-trehalase, acyltransferase, transferase, oxidoreductase, the photosystem electron transporter, glutathione transferase, hexokinase, chlorophyll catabolite reductase, and glucose binding were significantly enriched. KEGG analysis revealed significant enrichment in carbon metabolism, anthocyanin biosynthesis, chlorophyll metabolism, starch and sucrose metabolism, glycolysis/gluconeogenesis, glutathione metabolism, photosynthesis, pyruvate metabolism, galactose metabolism, α-linolenic acid metabolism, linoleic acid metabolism, glucosinolate biosynthesis, terpenoid backbone biosynthesis, and fatty acid metabolism pathways (Figure 3b).

### 3.3. Metabolome Analysis

**Figure 4:**
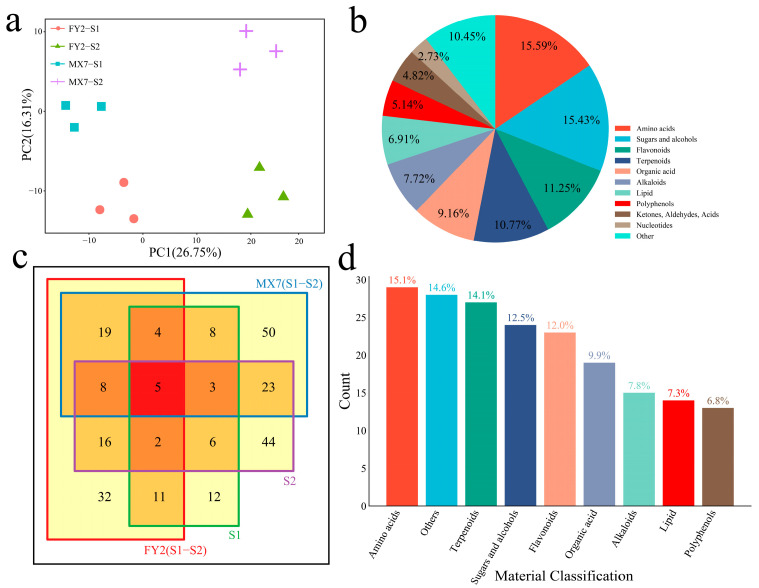
PCA and differential analysis of 12 strawberry metabolomes. (**a**) PCA of 12 strawberry metabolomes; (**b**) classification pie chart of metabolites detected in 12 strawberry metabolomes; (**c**) Venn diagram of DRMs between and within materials; (**d**) classification bar chart of DRMs between and within materials.

To study the differences in metabolites during strawberry fruit development, explore the changing characteristics of metabolites during development, and analyze the mechanism of fruit development, metabolome sequencing was performed on MX7 and FY2 fruits at two developmental stages. The PCA of the samples revealed that the distance between replicates in each developmental stage was small, indicating that the error between replicates was small and that the sample quality was good; thus, the next step in the analysis could be performed (Figure 4a). A total of 695 metabolites were identified during the two developmental stages of strawberry fruits. To understand the classification and functional characteristics of different metabolites, we classified and annotated the identified metabolites, and the highest contents consisted of amino acids (15.59%), sugars and alcohols (15.43%), flavonoids (11.25%), and terpenoids (10.77%) (Figure 4b). Finally, 243 differentially abundant metabolites were identified through differential expression analysis. A total of 97 DRMs, including 32 unique DRMs, were identified during the S1 and S2 periods of FY2, and a total of 121 DRMs, including 50 unique DRMs, were identified during the S1 and S2 periods of MX7 (Figure 4c). A total of 51 DRMs, including 12 unique DRMs, were identified during the S1 periods of MX7 and FY2. A total of 107 DRMs, including 44 unique DRMs, were identified during the S2 periods of MX7 and FY2 (Figure 4c). The DRMs primarily included amino acids (15.1%), terpenoids (14.1%), sugars and alcohols (12.5%), and flavonoids (12.0%) (Figure 4d).

**Figure 5:**
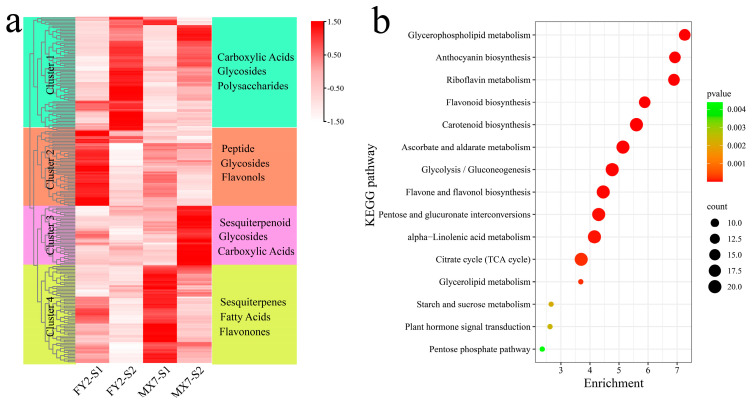
Clustering and KEGG enrichment analysis of DRMs between and within materials. (**a**) Clustering heat map of DRMs between and within materials; (**b**) KEGG enrichment analysis of DRMs between and within materials.

A total of four statistically significant clusters were identified for 243 differentially abundant metabolites via the k-means clustering method (Figure 5a). Cluster 1 had the highest content during the S2 period of FY2, which primarily included carboxylic acids, glycosides, and polysaccharides. Cluster 2 had the highest content during the S1 period of FY2, primarily consisting of peptides, glycosides, and flavonols. Cluster 3 had the highest content during the S2 period of MX7, with mainly sesquiterpenoids, glycosides, and carboxylic acids. Cluster 4 had the highest content during the S1 period of MX7, which was primarily comprised of sesquiterpenes, fatty acids, and flavonones. Metabolic pathway enrichment analysis of the differentially abundant metabolites was performed with the KEGG database, and the metabolites were found to be significantly enriched in glycerophospholipid metabolism, anthocyanin biosynthesis, riboflavin metabolism, flavonoid biosynthesis, carotenoid biosynthesis, ascorbate and aldarate metabolism, glycolysis/gluconeogenesis, flavone and flavonol biosynthesis, pentose and glucuronate interconversions, α–linolenic acid metabolism, the citrate cycle (TCA cycle), glycerolipid metabolism, plant hormone signal transduction, starch and sucrose metabolism, and the pentose phosphate pathway (Figure 5b).

### 3.4. Joint Analysis of the Transcriptome and Metabolome

**Figure 6:**
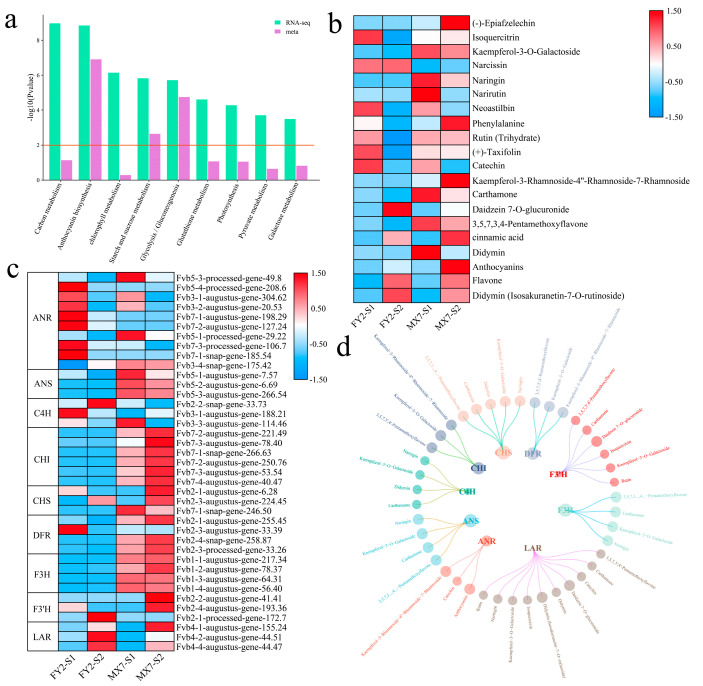
Joint analysis of DEGs and DRMs. (**a**) KEGG enrichment analysis of DEGs and DRMs; (**b**) Changes in the contents of metabolites in the anthocyanin biosynthesis pathway; (**c**) Heat map of the gene expression patterns in the anthocyanin biosynthesis pathway; (**d**) Correlation analysis between metabolites and genes in the anthocyanin biosynthesis pathway.

The KEGG pathway enrichment analysis highlighted significant enrichment of both DEGs and DRMs in pathways related to anthocyanin biosynthesis and starch and sucrose metabolism, as well as glycolysis/gluconeogenesis (Figure 6a). The alterations in metabolite levels within the anthocyanin biosynthesis pathway were assessed. (−)-Epiafzelechin, phenylalanine, kaempferol-3-rhamnoside-4″-rhamnoside-7-rhamnoside, cinnamic acid, and anthocyanins were the highest during the S2 period of MX7, and kaempferol-3-O-galactoside, naringin, narrutin, carthamone, 3,5,7,3,4-pentamethoxyflavone, and didymin were the highest during the S1 period of MX7 (Figure 6b). Daidzein 7-O-glucuronide had the highest content during the S2 of FY2, whereas flavone and didymin (ososakuranetin-7-O-rutinoside) had the highest content in the S2 of both materials. The gene ANR in the anthocyanin biosynthesis pathway exhibited its highest expression level during the S1 stage of FY2, which then decreased as fruit development progressed (Figure 6c). The ANS gene displayed the highest expression level in MX7, with its expression level showing a decreasing trend during fruit development. Among the C4H genes, *Fvb2-2-snap-gene-33.73* presented the highest expression in S2 of FY2; *Fvb3-1-augustus-gene-188.21* presented the highest expression during the S1 of FY2; and *Fvb3-3-augustus-gene-114.46* presented the highest expression in the S1 of MX7. CHI, CHS, DFR, F3H, and F3′H were all expressed at the highest levels in the S2 period of MX7, and their expression levels increased with fruit development. These findings indicate that the accumulation of anthocyanins in strawberry pulp is regulated primarily by the CHI, CHS, DFR, F3H, and F3′H genes. A network incorporating Differentially Expressed Genes (DEGs) and Differentially Methylated Regions (DRMs) within the anthocyanin biosynthesis pathway was established using screening criteria of a Pearson correlation coefficient (PCC) ≥ 0.95 and a *p*-value < 0.05. The analysis revealed that the LAR gene could regulate 10 metabolites in the anthocyanin biosynthesis pathway, while F3′H was found to regulate six metabolites within the same pathway (Figure 6d). In addition, CHS was demonstrated to be a key gene related to anthocyanins, cinnamic acid, and phenylalanine.

### 3.5. WGCNA

**Figure 7:**
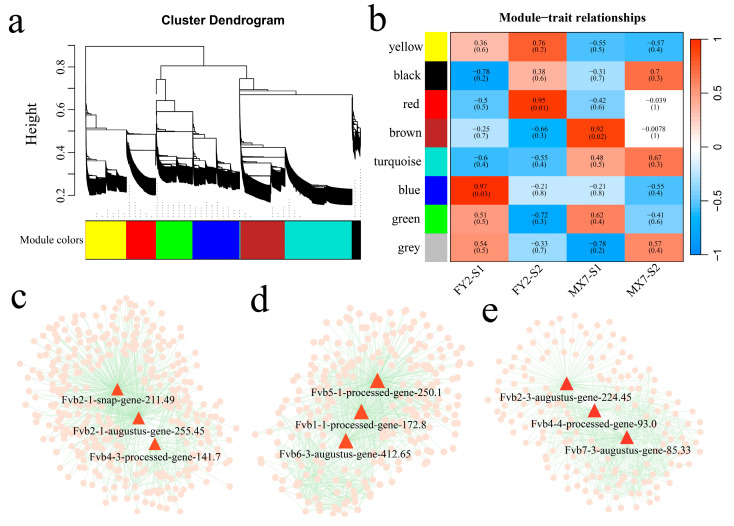
WGCNA. (**a**) WGCNA cluster dendrogram, with different colors representing different modules; (**b**) Correlations between modules and two developmental stages of strawberry pulp; (**c**) Red module gene network diagram; (**d**) Brown module gene network diagram; (**e**) Blue module gene network diagram.

Based on the expression of 13341 DEGs, β = 4 was selected to construct the network, and a total of eight co-expression modules were obtained (Figure 7a). The correlations between the modules and the two developmental stages of FY2 flesh and MX7 flesh were calculated (Figure 7b). The red module displayed a significant correlation with the S2 period of FY2; the brown module was notably linked with the S1 period of MX7; and the blue module exhibited a substantial correlation with the S1 period of FY2. By identifying the three genes with the highest connectivity in each module as hub genes, a total of nine hub genes were identified (Figure 7c). *Fvb2-1-augustus-gene-255.45* encodes the DFR protein; *Fvb2-3-augustus-gene-224.45* encodes the CHS protein; *Fvb1-1-processed-gene-172.8* encodes the MYB transcription factor; *Fvb4-4-processed-gene-93.0* encodes the bHLH transcription factor; *Fvb4-3-processed-gene-141.7* encodes the ERF transcription factor; *Fvb5-1-processed-gene-250.1* encodes sucrose synthase, a protein in the sugar metabolism pathway; *Fvb2-1-snap-gene-211.49* encodes β-1,3-galactosyltransferase; *Fvb6-3-augustus-gene-412.65* encodes cytochrome P450; and *Fvb7-3-augustus-gene-85.33* encodes the non-phototropic hypocotyl 3 (NPH3) protein.

### 3.6. qRT–PCR

**Figure 8:**
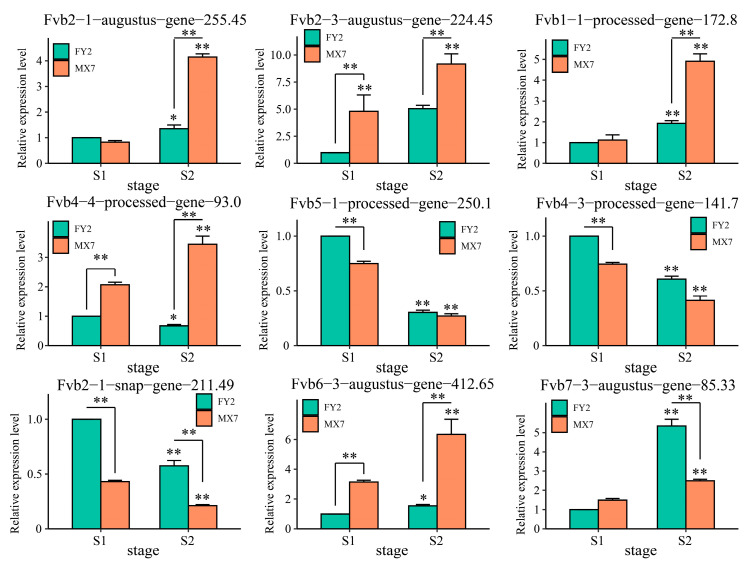
Expression patterns of nine candidate genes before and after the strawberry flesh color change. The results are presented as the means ± SDs (n = 3, * *p* < 0.05, ** *p* < 0.01).

The association between a gene’s expression pattern and its function is significant. Hence, the expression profiles of nine candidate genes were analyzed using qRT-PCR (Figure 8). The expression levels of *Fvb2-1-augustus-gene-255.45*, *Fvb2-3-augustus-gene-224.45*, *Fvb1-1-processed-gene-172.8*, *Fvb4-4-processed-gene-93.0*, *Fvb6-3-augustus-gene-412.65*, and *Fvb7-3-augustus-gene-85.33* increased significantly with the development of strawberry fruit. Among these genes, the expression levels of *Fvb2-1-augustus-gene-255.45*, *Fvb2-3-augustus-gene-224.45*, *Fvb1-1-processed-gene-172.8*, *Fvb4-4-processed-gene-93.0*, and *Fvb6-3-augustus-gene-412.65* in MX7 were greater than those in FY2, and the expression level of *Fvb7-3-augustus-gene-85.33* in FY2 was significantly greater than that in MX7 during the S2 period. The expression levels of *Fvb4-3-processed-gene-141.7*, *Fvb5-1-processed-gene-250.1*, and *Fvb2-1-snap-gene-211.49* decreased significantly with the development of strawberry fruit, and their expression levels during FY2 were significantly greater than those in MX7.

## 4. Discussion

As social advancements and enhanced human living conditions continue, consumer focus has shifted toward the quality of fruits and vegetables. Fruit color, being a key visual attribute, plays a fundamental role in the overall quality of the appearance of fruits and vegetables, directly influencing their economic value [40]. Varied breeding of fruits and vegetables serves not only to cater to diverse consumer preferences but also to enhance growers’ economic returns. Transformations in peel and fruit color are intricately connected to pigment biosynthesis, contributing to the production of red, purple, blue, and yellow-hued fruits [41]. The principal pigments influencing plant tissue color include anthocyanins and carotenoids. The synthesis and buildup of anthocyanins play a critical role in determining the vibrant red hue of fruits [42]. From the plant breeding perspective, changes in peel and flesh color are important goals. For example, dragon fruit with different flesh and peel colors and pineapple with light red spots can attract many consumers [43,44]. Studying the core pathways, genes, and metabolites involved in the process of strawberry fruit color transformation is highly important for strawberry breeding. Therefore, in this study, two strawberry varieties with different flesh colors, FY2 (white flesh) and MX7 (orange-red flesh), were selected for RNA-seq and metabolome analyses before and after color changes, and the correlations among the samples were evaluated. Cluster analysis and PCA revealed that the difference between the two MX7 stages was greater than that of FY2, and more DEGs and DRMs were identified during the S1 and S2 stages of MX7.

Sugars in the fruit are the main form of transport for leaf photosynthesis assimilates between the source and the sink, so the transport and metabolism of sugars play key roles in fruit development [45]. The photosynthesis of plant leaves results in the production of assimilates, which undergo metabolism and transmembrane transport under the action of related enzymes after long-distance transportation and ultimately accumulate in the fruit in the form of glycogen or other forms, resulting in the formation of different flavors [46]. Within strawberry fruits, three primary sugar types include sucrose, glucose, and fructose. Among these, fructose is perceived as the sweetest, followed by sucrose, while glucose is considered to have the lowest sweetness [47]. During the initial phases of fruit growth and development, it is significant to note that hexose sugars (comprising fructose and glucose) make up approximately 80% of the total sugar content. As strawberry fruits mature, the accumulation of fructose, glucose, and total sugar continues to increase. When the fruit enters the initial ripening and mature stages, sucrose accumulates rapidly [48]. Due to the varying nature of fruits, the types and quantities of soluble sugars differ. In the case of strawberry fruits, the rapid accumulation order for the three main soluble sugars is as follows: fructose, glucose, and sucrose [48]. Our investigation through RNA-seq and metabolomics analyses revealed significant enrichment of pathways associated with sugar metabolism, particularly in starch and sucrose metabolism, evident in both DEGs and DRMs. Notably, sucrose content witnessed a substantial increment during strawberry fruit development, with higher levels observed in MX7 compared to FY2. Detailed examinations of metabolites and genes within the sugar metabolic pathway in fruits hold great significance for enhancing the quality and yield of strawberries, with a potential heavy reliance on photosynthesis.

Anthocyanin synthesis in fruits follows the phenylalanine metabolic pathway and involves the cooperative action of specific genes, including structural genes like PAL and CHS. The expression levels of these crucial enzyme-encoding genes associated with anthocyanin synthesis are notably elevated during the later stages of fruit development, and they play a pivotal role in the accumulation of anthocyanins [49]. Studies in plums have shown that anthocyanin biosynthesis is promoted by the upregulation of genes such as PsPAL, PsCHS, PsCHI, PsF3H, and PsDFR [50]. Cyanidin-3-galactoside is the main component in apple and red pear fruits [51]. The primary anthocyanin compounds influencing the color of grape skin are peony derivatives and malvidin-3-O-glucoside [52]. In the process of dates transitioning to a red color, the main pigment accumulation components are malvidin-3-O-glucoside and delphinidin-3-O-glucoside [53]. The red color transformation in peppers is a result of chlorophyll degradation and the synthesis of carotenoids [54]. In this study, the anthocyanin content increased during the red color change in strawberries, and the anthocyanin content in MX7 was greater than that in FY2. Therefore, the accumulation of anthocyanins may be the main reason for the red color change in strawberries. An in-depth analysis was conducted on the network involving DEGs and DRMs within the anthocyanin biosynthesis pathway. This analysis showed that the LAR gene has the ability to regulate 10 metabolites within the anthocyanin biosynthesis pathway, while F3′H can regulate 6 metabolites in the same pathway. In addition, CHS was revealed to be a key gene related to anthocyanins, cinnamic acid, and phenylalanine. Flavonoid metabolites accounted for 12% of the DRMs. The KEGG enrichment analysis of DEGs and DRMs highlighted significant enrichments in anthocyanin biosynthesis, starch and sucrose metabolism, and glycolysis/gluconeogenesis pathways during the red color transition of strawberries. Hence, metabolites within these pathways play pivotal roles in the strawberry’s red color alteration process. Subsequently, breeding markers can be devised for the genes within these metabolic pathways, emphasizing the importance of screened key genes for future research focus. This study provides valuable insight into the strawberry pulp development mechanism.

The metabolism of plant anthocyanins is regulated by a variety of transcription factors, among which the MBW ternary complex with R2R3-MYB as the core and containing the bHLH and WD40 transcription factors is considered to play a major regulatory role [55]. Generally, the MBW complex primarily regulates the expression of related genes in the flavonoid metabolic pathway (DFR, ANS, LAR, ANR, UFGT, and GST), whereas the genes of the upstream phenylalanine metabolic pathway (CHS, CHI, F3H, F3′H, F3′5′H, PAL, C4H, and 4CL) are regulated primarily by the independent R2R3-MYB [56]. The *FaMYB9/FaMYB11-FabHLH3-FaTTG1* complex positively regulates the proanthocyanidin metabolic pathway in strawberries, whereas the MBW with *FaMYB10* as the core may positively regulate the anthocyanin metabolic pathway [57,58]. Moreover, *FaMYB1* inhibits the synthesis of strawberry anthocyanins by competing for bHLH binding sites in the MBW complex [59]. In addition, the *FaMYB5-FaEGL3-FaLWD1/FaLWD1*-like complex has been shown to simultaneously regulate the metabolism of proanthocyanidins and anthocyanidins in strawberries. Overexpressing *FaMYB5* notably enhanced the accumulation of proanthocyanidins and anthocyanidins in ‘Xiaobai’ and ‘Hongyan’ strawberry fruits. Conversely, transient silencing of *FaMYB5* resulted in a substantial reduction in anthocyanidin content in ‘Fengxiang’ fruits [60]. Studies have shown that the silencing of FvMYB10 in forest strawberries can lead to the blockage of anthocyanidin synthesis, whereas *FvMYB10* overexpression can significantly increase the content of anthocyanidins [61,62]. Similarly, transient silencing of *FaMYB10* in cultivated strawberries led to a significant decrease in anthocyanidin content, and transient overexpression of *FaMYB10* led to a significant accumulation of anthocyanidins in strawberries [63]. Moreover, the insertion of 8 bases (ACTTATAC), which leads to the loss of *FaMYB10-2* function, is considered the key factor causing the loss of anthocyanidins in the fruits of the cultivated ‘Snow White’ strawberry [64]. MYB10 alleles contribute to the inherent variability in strawberry fruit skin and flesh color. The insertion of the *FaEnSpm-2* transposon (CACTA) within the *MYB10-2* promoter primarily triggers elevated *MYB10-2* expression and enhances anthocyanin biosynthesis in strawberry flesh. In diploid wild strawberry (*Fragaria vesca*), *FveMYB10* is primarily responsible for regulating anthocyanin metabolism in strawberry red fruit, while its paralogous gene *FveMYB10L* is responsible for regulating anthocyanin synthesis in petioles, and the two genes play independent roles in fruits and petioles [65]. Other studies have shown that *FabHLH3*, a member of the MBW family, can bind to MYB-type transcription factors and positively and negatively regulate anthocyanins in strawberry fruit through the MBW complex [66]. In this study, we identified two new TFs by RNA-seq and qRT-PCR, namely, *Fvb1-1-processed-gene-172.8* (MYB) and *Fvb4-4-processed-gene-93.0* (bHLH).

## 5. Conclusions

In conclusion, in this study, we sequenced the transcriptomes and metabolomes of strawberries with two different flesh colors during the color change and ripening periods. The anthocyanin biosynthesis, starch and sucrose metabolism, and glycolysis/gluconeogenesis pathways were identified as important regulatory pathways for strawberry flesh color change via joint analysis of RNA-seq and metabolome data, and nine candidate genes related to strawberry flesh color were screened. This study provides a deep understanding of the dynamic transcription and metabolic regulatory network involved in strawberry flesh color changes. In fact, plant breeding programs rely on DNA-based molecular markers and may ignore many transcriptional and metabolic-level variations. Therefore, our study highlights the importance of transcriptional and metabolic levels, which is beneficial for future crop improvement strategies and technologies that consider not only genetic variation but also transcriptional and metabolic levels. The DEGs and DRMs associated with the process of strawberry fruit development and the candidate genes identified in this study may provide an initial set of basic data for the future expansion of research strategies. In addition, the specific regulatory mechanisms of other pathways, such as anthocyanin biosynthesis, starch and sucrose metabolism, and glycolysis/gluconeogenesis, in strawberry flesh development require additional experimental data for confirmation.

## Data Availability

The RNA data presented in this study have been deposited in the NCBI repository under the accession number PRJNA1157624.

## Supplementary Materials

The following supporting information can be downloaded at: https://www.mdpi.com/article/10.3390/genes15111391/s1, Table S1. All the primers used in this study. Table S2. Summary statistics for RNA-seq analysis of strawberry.
